# *Mycobacterium simiae*: Harmless colonizer or deadly pathogen?

**DOI:** 10.1371/journal.ppat.1008418

**Published:** 2020-04-30

**Authors:** Jean-Francois Jabbour, Amal Hamieh, Sima L. Sharara, Souha S. Kanj

**Affiliations:** 1 Division of Infectious Diseases, Department of Internal Medicine, American University of Beirut Medical Center, Beirut, Lebanon; 2 Division of Infectious Diseases, Department of Internal Medicine, Al Rassoul Al Azam Hospital, Beirut, Lebanon; 3 Division of Infectious Diseases, Department of Medicine, Johns Hopkins School of Medicine, Baltimore, Maryland; Nanyang Technological University, SINGAPORE

## What is the history of *Mycobacterium simiae* and what are some of its genomic components?

In 1965, a new species of nontuberculous mycobacteria (NTM) was isolated from Rhesus monkeys imported from India and was termed *Mycobacterium simiae* [1]. The name was derived from the Latin term *simiae*, which means “of monkeys” [2, 3]. *M*. *simiae* is a slow-growing photochromogenic mycobacterium that was initially considered an environmental pathogen mainly acquired from water [2, 4]. This organism can be found in municipal water sources [5], as well as soil, salt, foodstuff, and even air samples [6]. It has been identified in hospital drinking fountains, sinks, and ice machines and can act as a reservoir for nosocomial *M*. *simiae* outbreaks [2]. It can also contaminate medical equipment and laboratory specimens [3].

*M*. *simiae* is transmitted by inhalation of aerosols or by inoculation [7]. Human disease is attributed to environmental exposure to the pathogen, as there remains no evidence of human-to-human or animal-to-human transmission [8].

To date, there are only about 10 reported *M*. *simiae* strains that have been studied for genomic sequencing. The most recent strain, MsiGto, harbors housekeeping genes as well as virulence genes that confer infective mechanisms and host immune response evasion systems [9]. Some of these genes include *arcD*, an arginine and ornithine antiporter gene that can play a role in the persistence of the pathogen in host cells. *mce* operon clusters, which are common to most mycobacteria, are also found in *M*. *simiae*. Their role, mainly studied in *M*. *tuberculosis*, involves the secretion of Mce proteins that permit bacterial entry into mammalian cells and survival inside the macrophage. Mce proteins can also act as transporters and allow cholesterol degradation to free carbon and energy for use, which may facilitate the maintenance of infection. Contrary to other *M*. *simiae* models (DSM 44165 and MO323), MsiGto had an overrepresentation of the *mce3* cluster. Mce3 proteins are expressed in the infective phase of *M*. *tuberculosis*, and the acquisition of Mce proteins have been implicated in the transformation of some bacteria from an environmental to a pathogenic organism, such as in *Streptomyces* spp. [10]. Therefore, it is hypothesized that the presence of Mce proteins in *M*. *simiae* provides it with a pathogenic ability, even though many environmental mycobacteria possess them as well [9]. It is also postulated that organisms with a large *mce* copy number evolve pathogenicity faster than environmental mycobacteria [9].

Other genes include *fbpA*, *fbpC*, and *fbpD* (three out of four antigens from the 85 complex gene), which are not only responsible for cell wall synthesis but also encode enzyme products that play a role in the formation of Cord Factor (also known as trehalose dimycolate). This is one of the most important virulence factors of mycobacteria, as it has been linked to granulomatogenic activity in a tumor necrosis factor (TNF)-α dependent mechanism [9, 11]. However, the level of induction of TNF-α by the Cord Factor of *M*. *simiae* is lower than that of *M*. *tuberculosis* [11]. ESAT-6, a protein common to other pathogenic mycobacteria and which modulates immune responses by suppressing antigen presentation by the β-2 microglobulin chain of the major histocompatibility complex class I (MHC-I-β2M), is also found in *M*. *simiae* [9, 12].

## What are the epidemiology and regional distribution of *M*. *simiae*?

The prevalence of *M*. *simiae* varies by region. It has been isolated from many countries, with a notable regional prevalence in Cuba, the Middle East, and the arid regions of the southwestern United States (Texas, Arizona, and New Mexico) [13]. There have been increasing reports from western European countries (including Spain and France), eastern Mediterranean countries (like Iran and Lebanon), and Asia-Pacific countries, such as South Korea. The distribution of *M*. *simiae* within restricted geographic regions may highlight the importance of environment, temperature, and humidity in its physiology [4].

Most studies concerning the prevalence of *M*. *simiae* are limited to case series or small-scale studies. *M*. *simiae* accounts for 30% of all human potential pathogenic NTM in Israel in a 7-year period between 1975 and 1981 [14]. In Mumbai, *M*. *simiae* represented 35% from the slow-growing NTM, while *M*. *intracellulare* had the highest percentage (40%) [15]. Its rate varies between 0.8% and 15.3% in hospitals in Houston [2]. In Lebanon, *M*. *simiae* represents 30% to 65% of isolated NTM over the past two decades [4]. A recent meta-analysis from Iran found that the pooled national prevalence of *M*. *simiae* among NTM is 25% [16].

**Fig 1:**
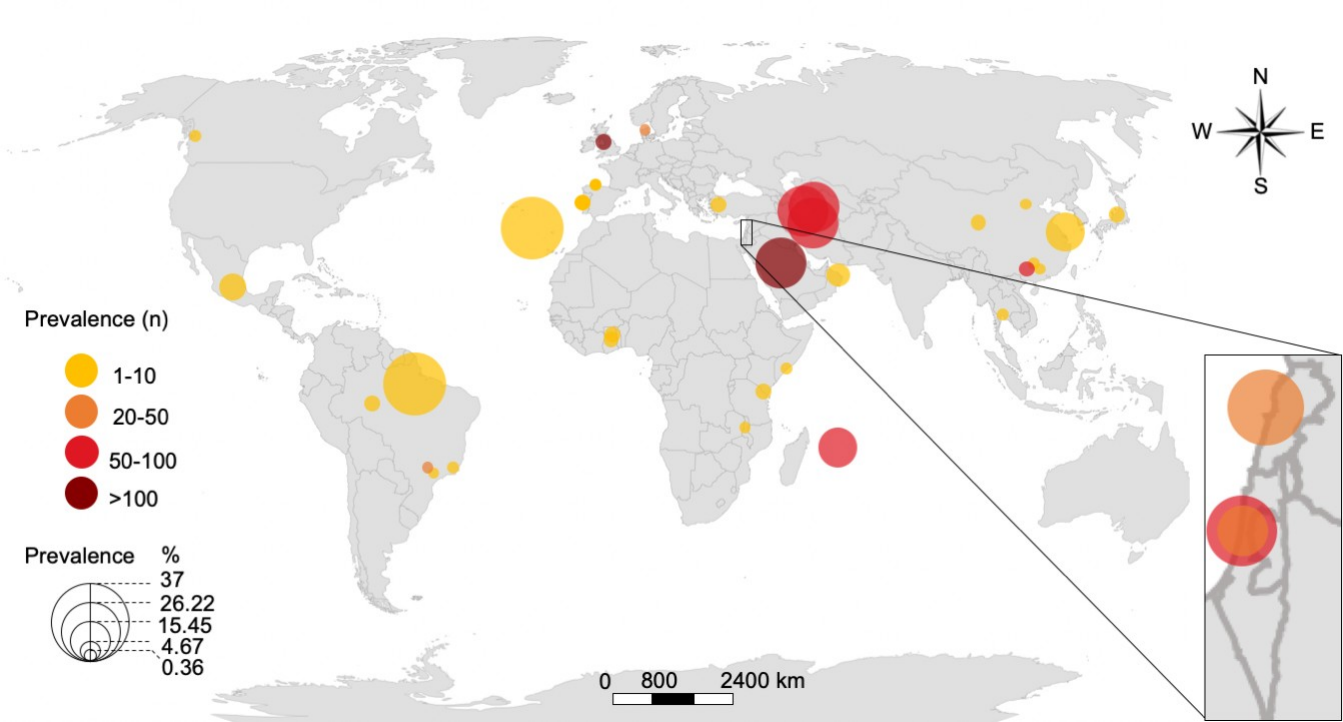
Geographic representation of the prevalence of *M*. *simiae*. Each dot represents a single study that has reported the prevalence (n) of *M*. *simiae* and the percentage of the species among NTM. Only studies that have reported both the prevalence and percentage among NTM were included. Sites included are: Brazil (Para, Sao Paolo, Rio de Janeiro, and Porto Velho), Canada (British Columbia), China (Beijing, Guangxi, Guangzhou, and Shanghai), Denmark (nationwide), France (Réunion Island), Ghana (East Mamprusi District and Tamale Metropolis), Iran (Tehran, Gorgan, and Ahvaz), Israel (Haifa and nationwide), Japan (Hirakocho), Kingdom of Saudi Arabia (nationwide), Lebanon (Beirut), Malawi (Lilongwe), Mexico (Mexico City), Mozambique (Maputo), Oman (Muscat and nationwide), Portugal (Lisbon), Spain (Asturias and Gran Canaria), Tanzania (Ngamiani and Makorora), Thailand (Bangkok), Turkey (Aegean region), and the United Kingdom (London). The map template is courtesy of GeoNames, HERE, Microsoft, NavInfo, Thinkware Extract, and Wikipedia and was created using Microsoft Excel and PowerPoint. n, number; NTM, nontuberculous mycobacteria.

The epidemiology is quite different between countries and may vary between centers from the same country (Fig 1). Fig 1 summarizes the existing literature on national *M*. *simiae* prevalence rates and percentages among NTM. It is worth mentioning that many developing countries do not routinely conduct NTM speciation, and therefore *M*. *simiae* could be underdiagnosed.

Along with geographical influence, *M*. *simiae* infections can also have a genetic predisposition. Individuals with genetic defects in the mononuclear phagocyte T-helper cells type 1 pathway can develop immunodeficiencies that cause mendelian susceptibility for mycobacterial diseases (MSMD). For instance, there is a report of two unrelated cases of disseminated *M*. *simiae* infections in children with interferon gamma receptor 2 (IFN-γR2) deficiency of consanguineous Arab and Israeli descent [17]. This is the only report that links *M*. *simiae* infections in the setting of MSMD with its geographical distribution.

## What is the clinical spectrum of *M*. *simiae* infections?

*M*. *simiae* was initially considered as an environmental pathogen and is rarely associated with human illness [4]. More recently, *M*. *simiae* has been described to cause a spectrum of clinical syndromes, ranging from an asymptomatic infection to a fatal disseminated disease [5, 18, 19].

*M*. *simiae* mostly causes pulmonary disease [5]. Immunocompetent patients usually develop respiratory infections in the setting of underlying lung diseases such as cystic fibrosis and chronic obstructive pulmonary disease, smoking, or a history of pulmonary tuberculosis [20]. Diabetes mellitus, cardiovascular disease, and malignancy are other risk factors associated with *M*. *simiae* pulmonary infection [21]. Patients typically present with nonspecific symptoms, including productive cough, hemoptysis, dyspnea, fever, night sweats, malaise, and weight loss [3, 21].

There have been increasing reports of disseminated *M*. *simiae*, particularly among immunosuppressed individuals such as patients with human immunodeficiency virus (HIV) [19, 22]. Almost all the reported HIV-infected patients have advanced acquired immunodeficiency syndrome. However, disseminated disease has also been described in an elderly otherwise healthy individual [8]. The presentation of disseminated *M*. *simiae* mimics infection with *M*. *avium* complex with systemic symptoms of fever, chills, malaise, and diarrhea and can be severe and fatal.

*M*. *simiae* can also cause various focal infections [20]. Cases of osteomyelitis in the spine, pelvis, and femur [5], genitourinary infections [5], lymphadenitis [6], meningitis [8], and skin and soft tissue infections [23] have been reported.

## Is it always alarming to isolate *M*. *simiae* from a culture specimen?

*M*. *simiae* infections can be severe, particularly in immunosuppressed patients, yet the isolation of *M*. *simiae* from respiratory specimens does not always represent true infection. Although advancements in laboratory diagnostics have enhanced the ability to detect *M*. *simiae*, it appears to have low pathogenicity. Most cases of *M*. *simiae* represent environmental contamination rather than clinical disease [8]. It is estimated that only 9% to 21% of *M*. *simiae* pulmonary isolates are clinically significant [3].

Treatment can be deferred in cases where *M*. *simiae* isolation is asymptomatic, as demonstrated in a study from Israel by Lavy and colleagues, in which *M*. *simiae* was isolated only once in more than 80% of asymptomatic patients that did not require treatment [14]. In a cohort of 97 patients with positive *M*. *simiae* cultures in the French Réunion Island, only 8% required treatment [18]. However, a recent cohort study from Lebanon [4], which reported a prevalence of *M*. *simiae* among NTM that was more than twice that of Réunion Island, reported a higher pathogenicity (47% versus 21.6% respectively) [24], suggesting a possible underestimation of the virulence of the organism.

In 2007, the American Thoracic Society (ATS) and the Infectious Diseases Society of America (IDSA) released guidelines to identify true NTM infection and distinguish cases of contamination or colonization with the organism. The guidelines recommend correlating a positive culture with clinical, microbiologic, and radiologic criteria while excluding other possible diagnoses to differentiate between true infection and colonization [8]. The isolation of *M*. *simiae* from sterile samples should always be considered clinically significant and dictate the need for antimicrobial therapy [19].

## What are the radiological findings of pulmonary *M*. *simiae* infection, and how can they help distinguish between tuberculosis and *M*. *simiae*?

*M*. *simiae* pulmonary infection can affect any lobe in the lungs and can be associated with lymphadenopathy, pleural effusion, and pleural thickening. Findings of *M*. *simiae* infection on chest X-rays are usually nonspecific [20]. Nodular lesions are the most common finding and are reported in up to 100% of patients [20]. Other radiographic findings include bronchiectasis (85%), tree-in-bud sign (88%), consolidation (53%), and lobar fibrosis or volume loss (67%) [20]. In addition, emphysema, chest wall deformities, and pneumothorax can be present.

In the lab, *M*. *simiae* is the only niacin-positive NTM and can be confused with *M*. *tuberculosis* [25]. Lung computed tomography (CT) scan findings, however, may be helpful to differentiate between the two entities [25]. Signs of chronic lung disease on chest imaging, particularly in the middle and lower lobes, are characteristic of *M*. *simiae*. Tuberculosis more often presents as cavitary lesions, usually with upper lobe involvement, along with bilateral disease and lymphadenopathy [25]. However, this clear-cut distinction does not always apply, as a recent study found no significant differences in pulmonary CT scan results between *M*. *tuberculosis* and *M*. *simiae* pulmonary infections [20]. It is important to distinguish between these two pathogens as the treatment regimens are different.

## Is infection due to *M*. *simiae* easily treatable?

The diagnosis of *M*. *simiae* infection does not immediately warrant the initiation of treatment. Physicians should weigh the risks of pharmacological burden according to the patient’s history and clinical setting before committing to a course of antimicrobial therapy [4, 8]. Similar to most NTMs, the majority of *M*. *simiae* isolates have an intrinsic or acquired resistance to first-line antituberculous medications. For example, (1) lack of prodrug activation, (2) polymorphisms in *embB*, *lfrA*, and efflux pump, and (3) ADP-ribosylation are intrinsic mechanisms of resistance to isoniazid, ethambutol, and rifampicin, respectively, that are commonly expressed in *M*. *simiae* [26]. Acquired resistance to isoniazid is through *katG* or *inhA* mutations, to ethambutol through mutations in *embB*, *embR*, and other genes in the *emb* operon, and to rifampicin through mutations in *rpoB* [26]. Furthermore, the treatment of *M*. *simiae* can be challenging since in vitro susceptibility results are not always correlated with in vivo susceptibility [5].

The 2007 IDSA guidelines on NTM suggest a treatment regimen for *M*. *simiae* similar to *M*. *avium* complex infection. A combination therapy based on macrolides is proposed [5, 8] with moxifloxacin, clofazimine, and streptomycin [8, 25, 27]. A drug regimen composed of clarithromycin, ethambutol, and ciprofloxacin has been reported to be successful in treating disseminated *M*. *simiae* infections in eight HIV patients [27]. Other macrolide combination therapies, such as adding clarithromycin to quinolones and trimethoprim and sulfamethoxazole (TMP/SMX), or ethambutol and TMP/SMX, have also been studied [8, 25, 27]. However, much like *M*. *abscessus*, some strains of *M*. *simiae* are resistant to macrolides through mutations in the *erm* gene or in the 23S rRNA [26]. There is a potential role for agents such as linezolid, cycloserine, or ethionamide in treating such infections [5, 25]. On the other hand, aminoglycosides have little to no role in the treatment of *M*. *simiae* infections, due to mutations in the 16S rRNA gene *rpsL* and aminoglycoside phosphotransferases or acetyltransferases that confer resistance to this class of antibiotics [9, 26].

There are no clear guidelines for the duration of therapy for *M*. *simiae* infections. In some reports, the treatment of pulmonary disease extended to more than 12 months after the first negative respiratory culture [4, 8]. The treatment of extrapulmonary disease is variable depending on the location of the infection. Surgery, such as debridement of a necrotic skin ulcer [23] or a laminectomy in vertebral osteomyelitis [5], in combination with antimicrobial therapy are clinically advised where appropriate [5].

## Is *M*. *simiae* a deadly pathogen?

The outcome of *M*. *simiae* infection is variable. Successful treatment of disseminated *M*. *simiae* infections has been described [27]. Patients with pulmonary disease could remain stable for many years. This was illustrated in a study of 102 patients, in which an excellent outcome was seen after a mean follow-up period of 24 months with no reported relapse or death related to the infection [21]. Other reports suggest improvement in some patients, relapse in others, or death, as seen in a case series from Lebanon [4]. There are reports of patient mortality early after diagnosis or after a few months of treatment [19].

The spectrum of *M*. *simiae* ranges from being an innocuous colonizer to a fatal organism, depending on the strain’s genetic composition, the patient’s risk factors and immunologic state, and the local epidemiology. This spectrum is a unique characteristic among NTMs, as most of them tend to be purely environmental or pathogenic. Advancements in diagnostic tools are facilitating the detection of *M*. *simiae*, yet there remains a marked paucity in the research related to the organism. A dedicated platform of research for *M*. *simiae* is necessary in order to gain more knowledge concerning its pathogenicity and to be able to differentiate its characteristics from other NTMs.
